# Figures That Feel and Work

**Published:** 1893-12-23

**Authors:** 


### Figures that Feel and Work.

There are figures and figures. In these pages, when we deal with figures, it is mostly our good fortune, or, as we sometimes feel it to be, our distressing ill-fortune, to deal with figures that breathe and live; that suffer from poverty and sickness, or from the agonies of accident and painful operations for relief or cure. Our figures are human creatures like ourselves, men, women, and young children, upon whom the Time-Spirit of hard fortune and pain has laid his unrelenting hand. For with all our advanced civilisation and the spread of science in all directions, we find that pain is not diminished either in its extent or its severity. On the contrary, the higher the civilisation, and the more general the education of man, the more sensitive he becomes to pain and suffering in mind, body, and estate.

### They Cannot Lie.

Figures can neither lie nor exaggerate. They are indeed very poor instruments for setting forth the full and living truth; but at least they have this advantage, that they do not set forth more than the truth. The figures tells us, for example, that there are eleven children's hospitals in London, and that these treat annually 6,086 in-patients, and close upon 80,000 out-patients. They do not tell us how many additional hospital beds are needed by the suffering children of the metropolis. But the absence of children's figures from the Westminster district and from Islington and the North-West, where there are no children's hospitals at all, shows us that at least one-eighth part of London has, up to the present time, made no provision whatever for its sick children. It comes, therefore, to this in practice—that the children of Islington and North-West London who are suffering from illness or accident must either be carried for miles to the children's hospitals of other metropolitan districts or endure the agonies, dangers, and disadvantages of nursing and treatment in their own poor homes. This ought not to be so. It is one of the facts which demonstrate the necessity for some central control of the London hospitals. The stoniest of hearts is softened and melted by the sicknesses, pains, and sorrows of children. A general hospital, even if it had room for them, which in Islington and the North-West it has not, is a painfully dull and depressing place for the very young. But a real children's hospital, strange as it may seem, is like a large and exceedingly lively nursery or play-room. The order of the day is "liveliness" for the children's sakes. The nurses are lively, and even the doctors; and the "merry heart" is found to promote recovery and strength in a very remarkable degree. The moral of this little digression is that it would be the dawn of a happy day for the children of Islington and North-West London if some kind-hearted philanthropist would build and endow at least one hospital in the north to be devoted to their exclusive use.

### The Woman's Era.

The close of the nineteenth century will probably be known as the "Woman's Era." Tennyson in his younger days told the world that "woman is the lesser man," and among the industrial poor woman certainly is not only the lesser, but very much the lesser man. The man does his day's work, and when it is over he has finished, and can play or improve his mind. But the mother of a working man's family, who is "cook, slut, and dairymaid" for the whole household; who is laundry and sewing maid into the bargain; and who is also chancellor of the exchequer; and all perhaps on twenty-five shillings a-week—when is her work done? When does she find time to play?

These working men's wives, these unspeakable char-women, and other female slaves—what would become of them when sick if there were no hospitals? To them five or six weeks in hospital must be like five or six weeks in heaven. Who is not glad to be told that London has fourteen hospitals for women, scattered about in its various districts? and that these hospitals provided for 5,630 in-patients, and gave treatment and medicine to 31,725 out-patients last year? In thinking over the subject one cannot but feel that modern life in large towns presses more hardly upon women of the working class than upon any other order of civilised beings. They are compelled to work after centuries of dependence, and they have less courage, less self-assertion, less market skill than any other class of workers; and so they are over-bargained, beaten, and crushed down until life is worse for them than any slavery that history has known. What wonder they sometimes drink, or do even things more disgraceful still! If any money given by hospital philanthropists may bring back a return in personal happiness, surely it is that money which is spent in providing a quiet haven of rest in sickness for women of the child-bearing, toiling, and toil-destroyed class.

### What Hospitals do for Producers.

Turn now to the working-man aspect of the hospital organisation. Working-men, it is said, have the future of our country in their hands. We do not altogether believe this; but, at least, the power of production with us will be to a large extent the measure of our greatness. Our power of production is measured by the capacity of our producers. The hospitals of London increase our productive power by probably at least an eighth every year by the services they render to the whole producing class. The acute and chronic sicknesses, and accidents of which working men are the victims, are recovered from in a third of the time in hospitals which recovery would take if the cases were treated in their own homes. This is not because hospital doctors are so markedly superior to private doctors, but because the nursing and surroundings at hospitals are in a high degree favourable to recovery, whilst the nursing and surroundings in working-class homes conduce almost entirely to continued illness or even to death. This means not merely an enormous diminution of suffering, but an unmeasurable saving of life, health, and producing capacity. Consider the work which the general hospitals of London alone did last year—the general hospitals, that is to say, as distinguished from the women's and children's hospitals already spoken of, and the various classes of special hospitals, infirmaries, dispensaries, sick asylums, and so forth, not yet mentioned. The general hospitals alone, which are attended by the pick of the working classes and by the poorer fringe of the middle and professional classes treated more than 50,000 in-patients last year, and 613,194 out-patients. In other words 1,000 severe cases a week sought shelter and medical care inside these hospitals, and more than 12,000 a week, or 2,000 a day, thronged their out-patient departments. It is a most striking spectacle. The 50,000 in-patients represented every variety of the most severe, dangerous, and prolonged illness, and the large majority of the 50,000 consisted of wage-earners and bread-winners. These are facts which appeal, not to sentiment merely, but to thought and the economic faculty. Whoever has the capacity for measuring economic and social factors on an adequate scale cannot but perceive how vast and valuable is the work represented by the figures 50,000 and 613,194 respectively with which we have just been dealing. But still more, whoever has the imaginative power which will enable him to trace the steps by which, from the smallest beginnings, the hospitals of the metropolis have attained their present dimensions, and their marvellous level of skill in medicine, surgery, nursing, and administration, will see reason to wonder more and more at the worth and greatness of that progressive civilisation which presents such institutions for our admiring gratitude and for the practical preservation of the life and health of our industrial classes.

### A Word for Specialisers.

A glance at one or two of the special hospitals will perhaps cause not a little surprise to those who have not previously acquainted themselves with the marvels of the medical work of philanthropic London. The diseases of the eye are very properly made the subjects of special study by a special class of men; and whoever reads carefully the series of articles now appearing in our columns by Mr. Brudenell Carter, F.R.C.S., will see how essential such a line of study is to the progress of medicine and the preservation of the people's sight. The hospitals for diseases of the eye alone treated last year as many as 2,599 in-patients, and the enormous total of 55,308 out-patients, in addition to the very large number of cases of eye disease which gravitated to the general hospitals. It is marvellous but most true, that so late as the beginning of the present century, and even later, practically nothing of a really scientific kind was known about the diseases of the eye. The best European surgeons fifty years ago were almost as ignorant and as barbarous in their methods as the Indian oculists who were prosecuted but the other day. Now, thanks to special eye hospitals and oculists, every deepest and minutest part of the eye stands revealed to science, and thousands of human beings every year are saved from blindness who, at the beginning of the century, would have been doomed to grope their way sightless through the world to the very last day of mortal life. If modern medicine had achieved nothing but its perfect mastery of diseases of the eye and of vision, it would still deserve the profoundest gratitude of the whole modern world. Of the other special hospitals which do excellent and necessary work, we cannot do more than make cursory mention. There are, as is well-known, special hospitals for the diseases of the throat and ear, for the skin, for cancer, for fistula, maternity, and orthopædic hospitals, epileptic and dental, Lock hospitals and Magdalene's homes. All these classes of institutions are doing indispensable work; and although we hope to see the day when most of the specialisms will be worked as departments of general hospitals, yet so long as the special hospitals continue to exist, and to do their work so excellently, we hope every one of them which has proved its merits to the satisfaction of those most competent to judge, will receive the steady and unwearying support of a discriminating charitable public.

### The Distribution of Medical Aid.

According to returns—which are perhaps as nearly exact as they could possibly have been made—the whole of the metropolitan hospitals, not including poor law infirmaries and fever hospitals, treated in the year ending December 31st, 1892, the grand total of 78,172 in-patients and 1,194,119 out-patients. Of these the South District, including Newington, received 14,126 in-patients and 166,228 out-patients; the East Central and City District, 6,899 in, and 142,773 out; the Westminster District, 10,465 in, and 169,767 out; the Marylebone and West Central District, 10,182 in, and 179,262 out; the Kensington and West District, 14,180 in, and 186,321 out; the Islington and North-West District, 7,008 in, and 133,417 out; and the Stratford and East-end District, 15,132 in, and 216,351 out-patients. The figures are easily written and easily read. But when it is remembered that every single unit represents a human being, and a human being who was present at the hospital because he was suffering and sick, the enormous total of human misery relieved, of human injury repaired, and of human happiness restored in that one single year, is overwhelming.

### The Money and the Money's Worth.

The figures which represent the money cost of all this work are easily set out; though here again the labour, the anxiety, and sometimes even the shifts and the straits to which the collectors of the money have been put in its collection would cause many a smile if they could all be recorded, and in some cases, perhaps, not a few heartaches. We deal with results and with large totals, and so also do our readers; but the hospital officials and workers deal with daily details, and these daily details are often nothing but daily struggles, daily anxieties, and daily disappointments. But, thanks to untiring energy and to a truly admirable amount of quiet and conscientious duty, the work is done, and done effectively, day by day, as well as year by year; and it is out of the million littles of the daily work that the magnificent yearly totals are made up.

The total sum expended last year in the treatment of the 78,172 in-patients and 1,194,199 out-patients was £677,724, an amount of which any city, however imperial, could hardly fail to be proud. Unhappily it was not enough, for more could have been spent at many hospitals to the great advantage of the public. But although the sum spent was not sufficient to overtake the actual necessities of the needy and deserving, it was much more than the hospital authorities found themselves actually able to collect. The total amount collected during the year was only £505,843. When this is compared with the £677,724 expended, a deficit is revealed amounting to the vast total of £171,881. At this point we stop. The hospitals cannot do less, and they cannot spend less; they ought to do more, and to spend more. How can this be accomplished with an annual deficit of £171,881? It remains for the wealthy and generous public to take this question seriously to heart, and to bring up the income of our noble medical charities to the level of the necessities of those whom they serve.

